# Enhancing land suitability assessment through integration of AHP and GIS-based for efficient agricultural planning in arid regions

**DOI:** 10.1038/s41598-025-14051-7

**Published:** 2025-08-26

**Authors:** Mohamed A. E. AbdelRahman, Taher M. H. Yossif, Mohamed M. Metwaly

**Affiliations:** 1 Land Use Department, Division of Environmental Studies and Land Use, National Authority for Remote Sensing and Space Sciences (NARSS), Cairo, 11769 Egypt; 2 Pedology Department, Water Resources and Desert Soils Division, Desert Research Center, Cairo, Egypt; 3 Data Reception, Analysis, and Receiving Station Affairs Division, National Authority for Remote Sensing and Space Sciences, Cairo, 11769 Egypt

**Keywords:** Land suitability, AHP, GIS, Land management, Crop rotation, Arid region, Climate sciences, Ecology, Environmental sciences, Environmental social sciences

## Abstract

Land Suitability Assessment (LSA) aids in identifying optimal crop cultivation sites; thereby, it is the key factor for proper planning to maximize production yield. It needs a combination of Analytic Hierarchy Process (AHP) and Geographic Information System (GIS) to improve LSA for the production of barley, beans, maize, soybean, sugar beet, and wheat in Egypt’s New Delta. Topography (slope), and characteristics of the soil (depth, pH, texture, carbonate content, and salinity) were the six factors employed. Land was assessed on a five-level suitability scale—highly suitable (S1), moderately suitable (S2), marginally suitable (S3), currently not suitable (N1), and permanently not suitable (N2). Pairwise comparisons and consistency ratios in AHP were used to determine weights for the criteria. Weighted overlay analysis produced land suitability maps. Key findings indicate slope as the primary factor for barley and wheat and soil properties as more significant for beans, soybean, and sugar beet. Barley, beans, maize, soybean, and wheat were assessed as highly suitable (S1), moderately suitable (S2), and marginally suitable (S3), but sugar beet was assessed as moderately suitable (S2). The results are valid for crop rotation for increased production and soil fertility by the appropriate use of AHP and GIS in sustainable land use management.

## Introduction

Agriculture is one of the cornerstones of Egypt’s economy and determinants of food security, particularly in light of Egypt’s fast-growing population. To address these challenges, Egypt has initiated an agricultural development strategy aligned with Egypt Vision 2030 and United Nations Sustainable Development Goals. They target optimizing utilization of agriculture resources, application of advanced farming methods, and expansion of sustainable land management to avert challenges such as food shortages, water shortages, and global warming^1^. The New Delta megaproject in the west Nile Delta is arguably the best plant in the proposal. The mega project is located on more than half a million hectares of reclaimed land for cultivation of strategic crops like wheat, maize, pulses, oilseeds, and vegetables. Besides cultivation, the project also includes the establishment of urban settlements with sophisticated administrative systems for efficiency and sustainability^2^.

The New Delta region has geographical advantages of a sort. Recently restored land with low fertility is well utilized to cultivate diversified crops such as barley beans, maize, soybeans, sugar beet, and wheat. Access to the Nile and the available irrigation equipment also guarantees there is water in Egypt throughout the year in the dry climate^3^. The scheme has a long-term influence on food security in the nation because it raises farm output and employment and hence results in economic growth and crop diversification^4^.

Ecological sustainability is the topmost priority of the New Delta project. With the help of advanced techniques like the Analytic Hierarchy Process (AHP) and Geographic Information System (GIS), the project enables sustainable land management measures to land degradation, soil fertility management, and climatic resilience^5^. Second-order land suitability analysis enabled by these techniques make way for long-term farm planning by organizing optimal patterns of crops and guiding sustainable land use^6^.

Land Suitability Analysis (LSA) is perhaps the most advanced planning technique for sustainable agriculture. LSA examines to what degree properly defined land units are suitable for cultivation under current environmental and management conditions^7^. By delineating land into suitability classes, e.g., highly suitable (S1), moderately suitable (S2), marginally suitable (S3), and not suitable (N), LSA provides a rational guidance to land use planning. Some of the most common LSA approaches are FAO and Sys et al.‘s parametric approach that have been applied very widely in agricultural development^8–10^.

New directions for LSA approaches have been offered by combining GIS with Multi-Criteria Decision Analysis (MCDA) approaches such as AHP. GIS facilitates simple visualization of data and spatial analysis, whereas AHP allows us to give relative weights to varying criteria, i.e., topography, soil class, and climate^11–13^. Saaty’s AHP approach, initially proposed by Saaty^14^, is appropriate in complicated decision-making scenarios and resolves them by the method of pairwise comparison and tests of consistency ratio^15^. These hybrid methods have also been utilized to great advantage for the purposes of improving land use planning, such as agricultural suitability mapping^16–18^.

GIS and MCDA were combined for ranking and mapping the allegedly suitable lands for agriculture^19–22^. GIS methods are employed in mapping the information, while in AHP weights are distributed among each factor such that key variables like climate, topography, and soil type get their rightful share in land suitability choices^23–25^.

Land suitability analysis earlier studies have only utilized soil data and developed derived suitability maps with no consideration of the other remaining determining factors of land suitability, such as topography and climate, and their interaction effects on one another. Omitting these entities in their models restricts them to be applied and used in diversified agricultural environments. Closing such gaps in research in this study, this paper combines GIS and AHP computer applications to offer an extensive, multi-dimensional system of soil appreciation. Utilizing topography, climate, and soil properties in the appraising process, not only does this study render more inclusive land suitability appraisal, but it also develops the most suitable crop rotation cycles for guaranteeing sustainable land resource management. This novel approach is a step ahead in agriculture planning and a step in the direction of sustainable development process in semi-arid areas.

The emphasis of this study is to formulate an efficient method for appropriate land suitability analysis for cultivation of chosen key crops based on consideration of the combined AHP and GIS process. The study also attempts to explore the optimal suitable crop rotation scheme for planning sustainable land use. Through the use of thematic and local suitability mapping, the output will be a land use planning input which will direct planners and policymakers towards supporting the achievement of sustainable agricultural development in semi-arid areas.

## Materials and methods

###  Description of the study area

**Fig. 1:**
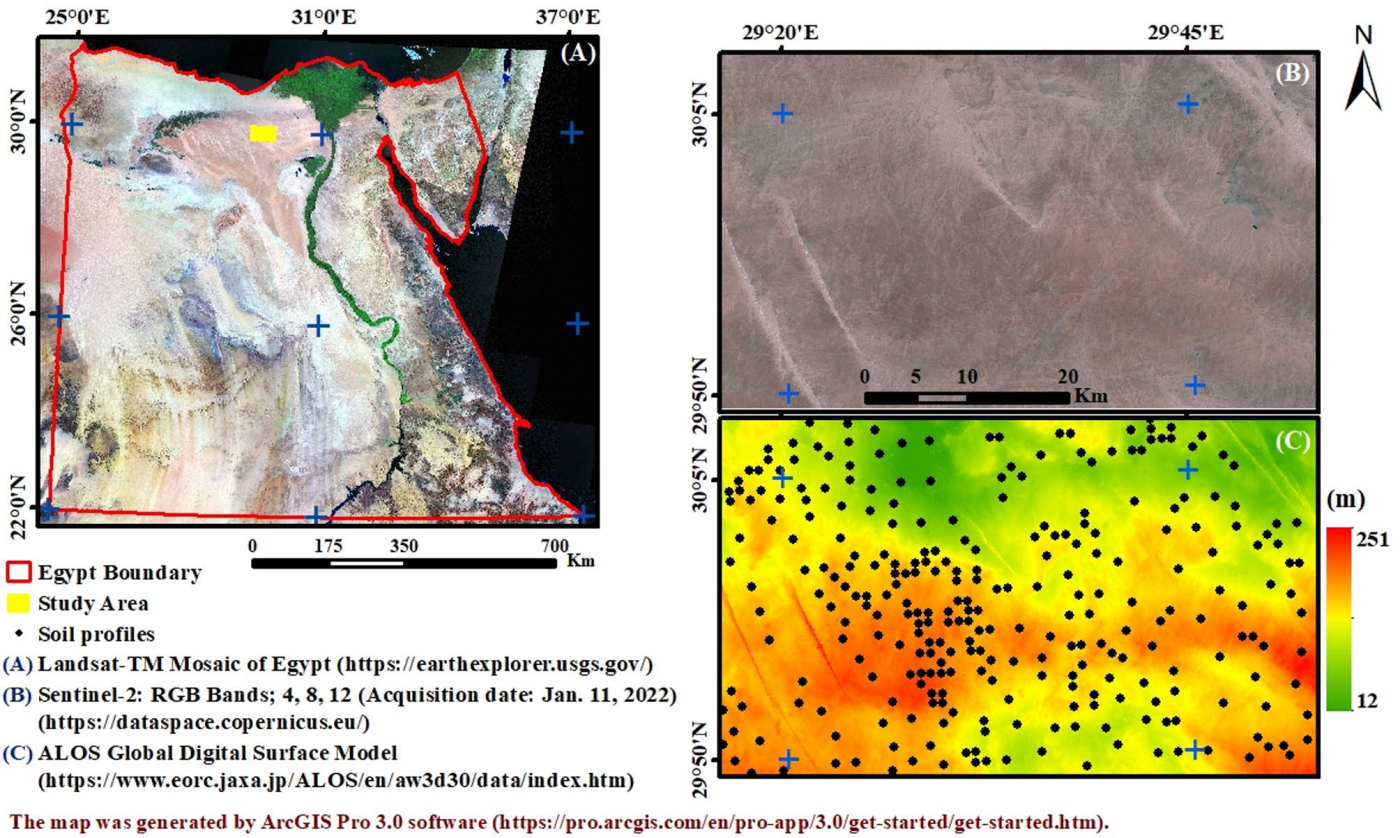
Location of the study area and soil profiles.

The study area is located in the Western Desert in Marsa Matrouh province between longitudes 29° 16” 10’ and 29° 52” 34’ and latitudes 29° 48” 50’ and 30° 8” 40’ with an area of 217,000 hectares (Fig. 1). The study area is included in the new Delta project. Based on the Digital Elevation Model (DEM), the elevation ranges from 12 to 251 m a.s.l. The region is designated as a newly reclaimed area in accordance with the state’s agricultural development goals. Groundwater serves as the primary water resource in the region, and alongside the prospective use of treated agricultural wastewater, it is being considered for the purpose of cultivating the newly reclaimed land.

**Fig. 2:**
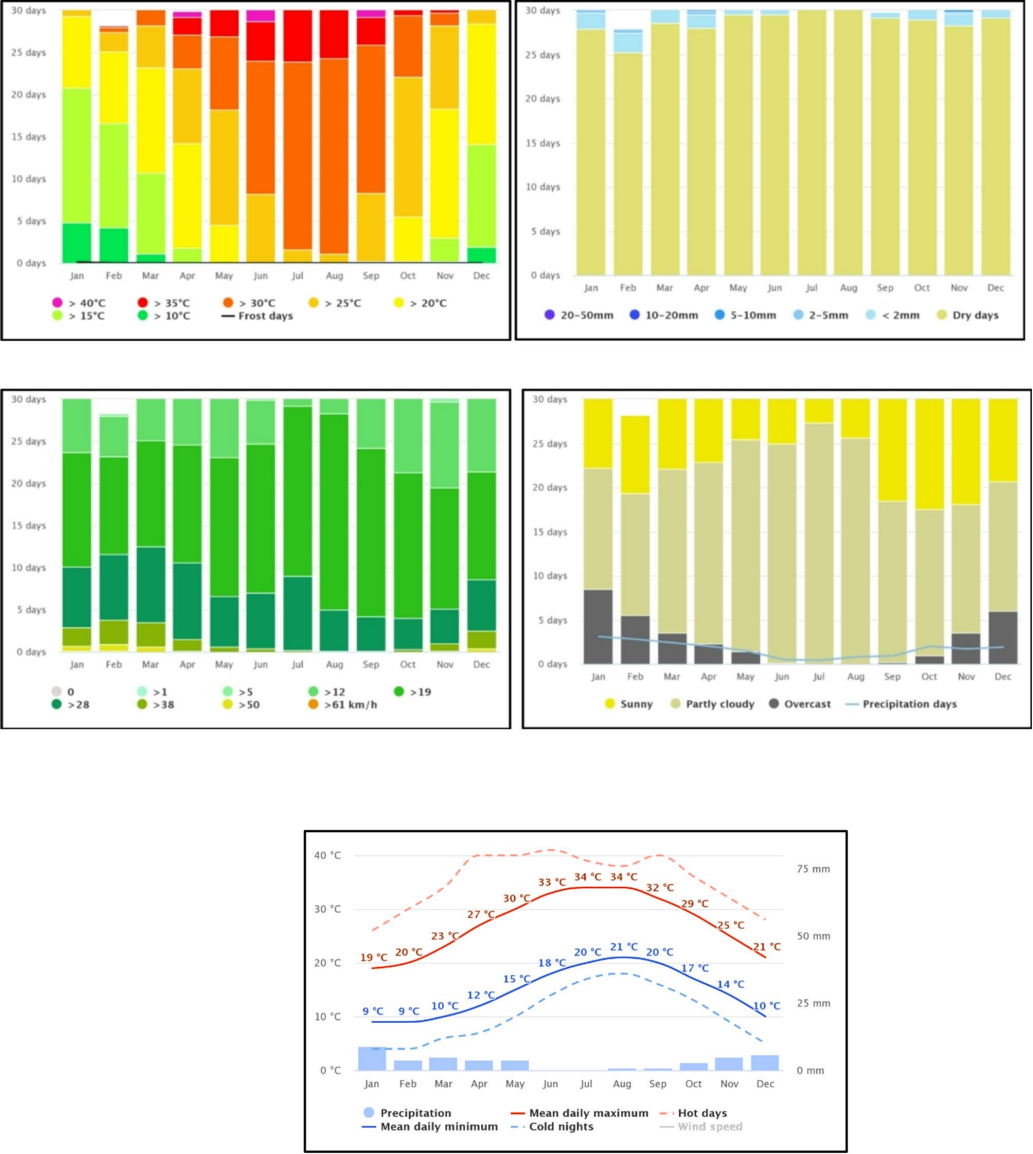
Climate diagrams based on 30 years of hourly weather model simulations for the years 1990 to 2020.

According to the Köppen climatic classification, the region has a dry-summer hot desert climate; however, winds from the Mediterranean Sea substantially reduce the temperature, making its summers moderately hot and humid and its winters mild and moderately wet. While the colder months have rain and cloud cover, the summers are sunny and dry. In the winter, sleet and hail are frequent (Table 1; Fig. 2).

**Table 1:**
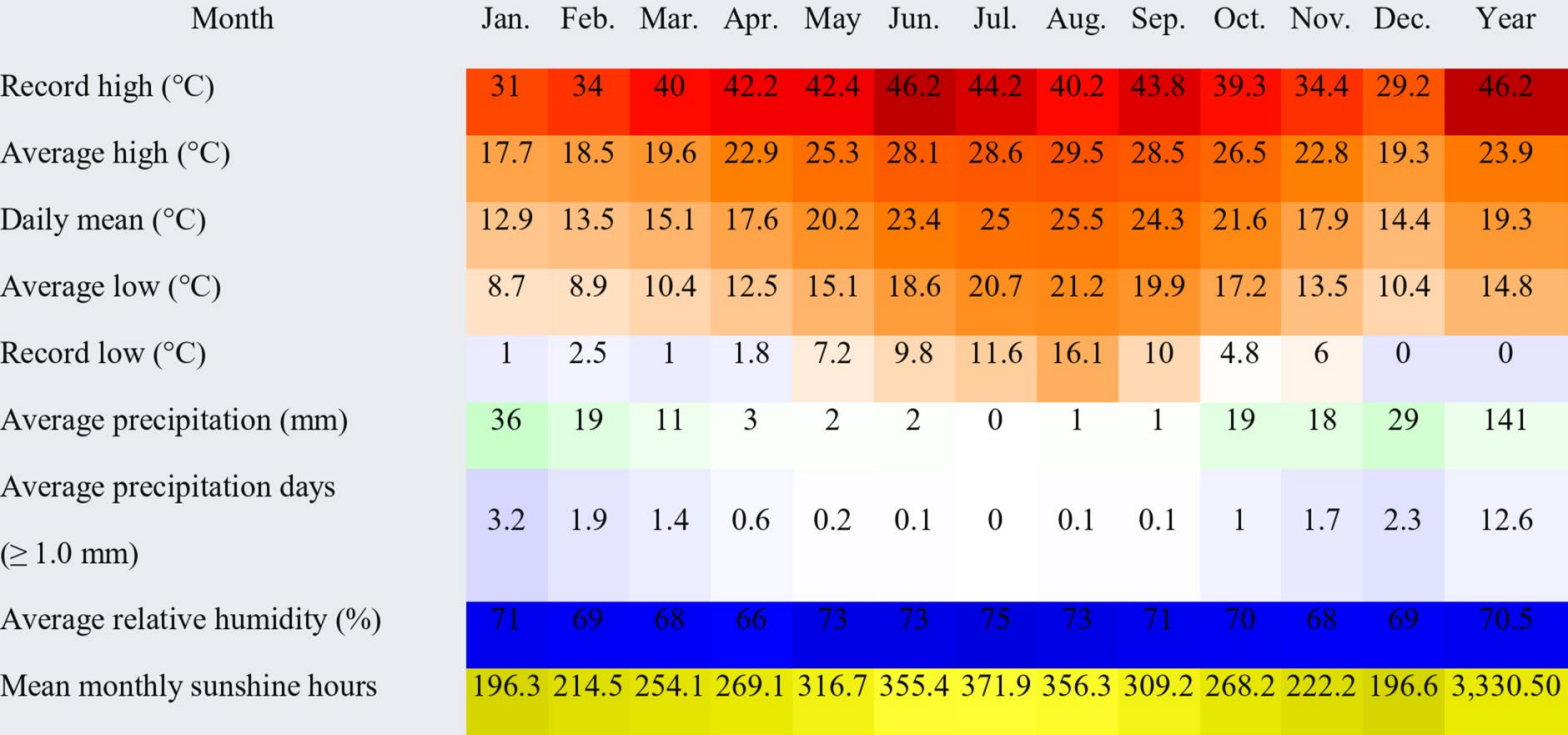
Climate data for the study area (average of the years 1990–2020).

Source: Egyptian meteorological authority.

### Topography data

In this study, the DEM-ALOS Global Digital Surface Model, downloaded online from https://www.eorc.jaxa.jp/, with a 30 m grid cell spatial resolution, was used to calculate the slope (as a percent) variable through the terrain analysis model in SAGA-GIS software version 9.4.1 (System for Automated Geoscientific Analysis).

### Field survey, sampling, and laboratory analyses

The field survey was carried out in January 2021, and the sampling procedure’s survey route was planned based on how easily accessible possible sampling sites were. Its geographic coordinates were captured using GPS. The soil sampling locations were allocated using a random distribution method in order to account for the variability in soil-landscape characteristics. 234 soil profiles were used to obtain soil samples. Using the Kellogg Soil Survey Laboratory Procedures Manual, chemical and physical studies were performed on the soil samples gathered from each of the genetic horizons or layers of the profile pits^26^.

The physical and chemical properties of the soil (soil depth, CaCO_3_, soil texture, salinity ECe, and alkalinity pH) are stored in shape file format. Therefore, soil properties are converted to raster format using Inverse Distance Weighted interpolation (IDW) in ArcGIS Pro 3.0 software, as recommended by AbdelRahman^27^.

### Land suitability assessment framework

**Fig. 3:**
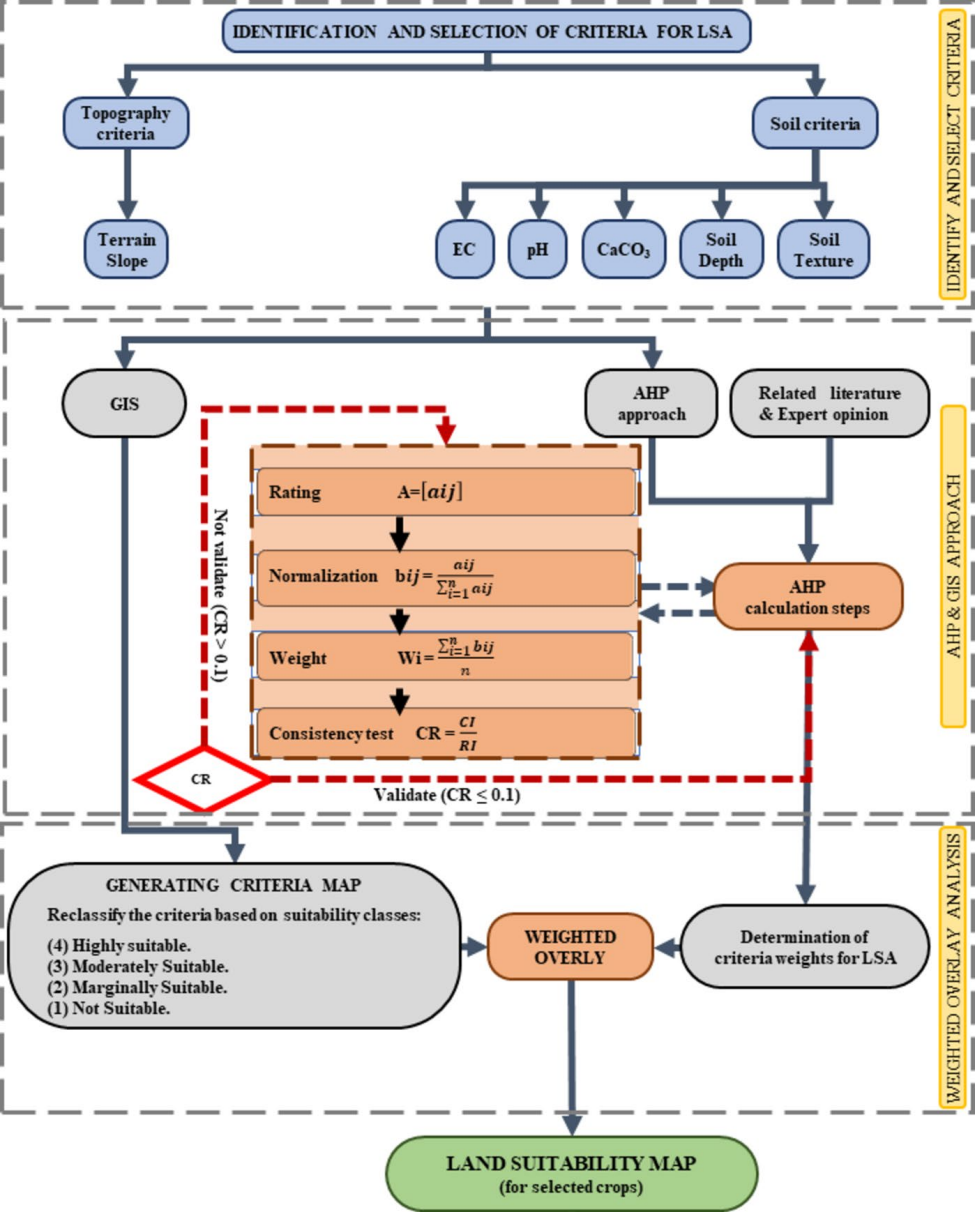
Flowchart illustrating the framework of land suitability modeling and weighting of criteria based on the AHP-GIS approach for land suitability assessment of selected crops.

Using the AHP-GIS based approach, the framework for LSA concerning specific crop cultivation can be summarized as follows: First, identify and select criteria that may affect crop growth and production; second, map these criteria using field data in a GIS environment using ArcGIS Pro 3.0 software; and finally, compare the criteria to determine their relative degree of importance using AHP in the Microsoft Excel Sheets software (Fig. 3). Finally, using a weighted overlay in ArcGIS Pro 3.0 software, the maps were combined and reclassified following the suitability levels. For this particular study, the following crops were chosen and evaluated: barley, beans, maize, soybean, sugar beet, and wheat. The selected crops are considered strategic crops in relation to ensuring food and water security and confronting the growing population, as stated in the national agricultural development goals outlined in Egypt’s Vision 2030 and the UN Sustainable Development Goals.

#### Identification and selection of criteria

The identification and selection of soil assessment for major crops was based on criteria that favour crop growth and production in arid conditions. The selected criteria were topography represented by land slope and various soil properties such as soil depth, pH, texture, carbonate and salinity.

In establishing these criteria, we used a pairwise comparison technique that involved the following: criterion establishment and ranking, through which the ranking and weighting of the criteria could be determined according to their relative importance; and review of the literature and established methodologies, focusing on studies related to agricultural practices in arid and semi-arid regions.

- Application of AHP: AHP was used to calculate the weights and ensure consistency in our choice of criteria.

The influential parameters in the assessment of soil evaluation for crops were identified and the standard range for the criteria was identified from the relevant literature. Each criterion was weighted based on these standard value ranges which are essential for any effective regional agricultural planning.

The objective of this study is therefore to explain the rationale for choosing the appropriate factors, methods and testing instruments and not to explain precisely everything that is done to test them.

Commonly used instrument for testing the named properties in soils:

Instruments for testing soil properties:

Soil depth: an auger, tap or corer. This allows samples to be taken from different depths for analysis.

Soil pH is measured using a pH meter or pH test kits. A soil sample is mixed with distilled water and then the pH meter or test kit measures the hydrogen ion concentration to determine the acidity or alkalinity of the soil.

-Soil texture: by the hydrometer method or sieve analysis, which separates soil particles into their sand, silt and clay fractions to obtain the texture class.

Soil carbonates: measured by calcimetry. This is a device that quantifies the carbonates in a soil sample by acidification with HCl and measures the volume of carbon dioxide produced.

The soil salinity level is measured using an EC meter, which is an instrument that measures the ability of the soil to conduct electric current; it is therefore directly related to the salt concentration in the soil. Each of these instruments provides valuable data for the overall assessment of the suitability of the land for crop growth.

##### Topography criterion (slope)

Topography, or relief, is one of the soil formations factors that significantly affect the distribution of soil properties. This factor influences soil genesis (i.e., drainage, runoff, deposition, and erosion, as well as the collection of solar energy). As a result, the topography factor is one of the key predictor variables in the prediction of soil properties^28^. The surface slope is one of the criteria that compose the topography factor (elevation, slope, and aspect), in which topography and the state of soil development are closely related. When selecting cropland, slope analysis is taken into account to help identify sites that could be suitable for agriculture purposes, afforestation, and watershed management. Slopes strongly influence the distribution of soil qualities, i.e., soil texture, soil depth, soil moisture, and the availability of nutrients and minerals. Deep soils are found in the gently sloping land at the bottom of valleys and foothill zones, whereas thin soils are dispersed on steep slopes with a higher level of erosion^17,29,30^.

The slope has an adverse effect on irrigation practices and mechanization, as high slopes increase the risk of erosion and cause the removal of nutrients and organic matter from the soil. Therefore, the slope criterion is considered a limiting factor for LSA for crops^17,31,32^. The suitable slope ranges vary for cultivating each crop. A slope less than 5 degrees is acceptable, and slopes between 5 and 15 degrees are thought to be moderately suitable. slopes of more than 30% (16.7 degrees) are classified as unsuitable for crop cultivation and machine operations^15,33,34^.

##### Soil criteria

1. Effective soil depth.
  Soil depth is one of the essential criteria of many land evaluation systems^7,20,35–38^; and is a factor in LSA^31^. Soil depth affects numerous soil qualities, i.e., the amount of soil nutrients, maximum water holding capacity, level of moisture, rate of infiltration, and plant growth, as well as agricultural productivity. Farmers choose their cropping patterns based on the slope and depth of the soil^25,29^.
2. Soil texture.
  Soil texture is used as a criterion in LSA due to its effect on many soil properties, i.e., soil structure, infiltration rate, water holding capacity, soil salinity (EC), soil alkalinity (pH), maximum water holding capacity, and soil nutrients, i.e., N, P, K, and OM. Therefore, many studies have used soil texture in LSA ^15^.

3. Soil reaction (pH).
  pH (soil reaction) is an effective criterion that influences nutrient availability, plant growth as well as productivity, the solubility of toxic ions, and microorganism activity^29,31^. Also, directly or indirectly, pH affects many physical, chemical, and biological activities in the soil. High pH values have been found to decrease the movement of phosphorus (P) and trace elements (Fe, Mn, and Zn) in the soil, and acidic soil has been found to increase the availability of hazardous substances to plants^17,25,31^.

4. Soil electrical conductivity (EC).
  EC is regarded as an indicator of the ion concentration of the soil solution^39^. If the EC rises above the essential levels for plants, which is directly related to soil fertility, vegetation decreases and plant growth is inhibited in subsequent seasons. The nutrient imbalance causes a decrease in nutrient intake, including P and other nutrients, as a result of high concentrations of Na^+^ and Cl^−^ under salty circumstances^17,31^.
5. Soil calcium carbonate (CaCO_3_).
  Soil CaCO_3_ content has no direct effect on crop yield or quality; it is stated that in calcareous soils phosphorous, forms compounds with Ca and, as a result, plant phosphorus uptake decreases^31^.

#### Standardization of criteria

For LSA, the criteria were standardized (normalized) in ArcGIS Pro 3.0 software according to the five levels of a framework for land evaluation^4^ and are as follows: highly suitable (S1), moderately suitable (S2), marginally suitable (S3), currently unsuitable (N1), and permanently unsuitable (N2). However, in this study, the last two levels (N1 and N2) were combined^21,40^. Standardization (normalization) scores of 4, 3, 2, and 1 were assigned for the four suitability classes (S1, S2, S3, and N), respectively, for all criteria. The rating of criteria for the selected crops (crop requirements) was proposed and adapted (Table 2) after^41–45^ and various sources. All raster-based maps for each criterion were reclassified using these scores and defined the suitability classes.

**Table 2:** Criteria reclassification and Punctuation for land suitability assessment for selected crop cultivation.

	Criteria	Suitability Classes			
		Highly Suitable (S1) (4)^1^	Moderately Suitable (S2) (3)^1^	Marginally Suitable (S3) (2)^1^	Not Suitable (*N*) (1)^1^
Barley	Slope %	< 8	8–12	12–16	> 16
	Texture	C < 60s, Co, SiCs, SiCl, Si, SiL, CL, C < 60v, SC, C > 60s, L, SCL, SL	C > 60v, LfS, LcS, fS, cS	-	Cm, SiCm
	Soil depth (cm)	> 50	50 − 20	20 − 10	< 10
	CaCO_3_%	< 30	30–40	40–60	> 60
	pH	7.2-8.0	8.0-8.2	8.2–8.5	> 8.5
	EC_e_ (dS/m)	< 12	12–16	16–20	> 20
Beans	Slope %	< 4	4–8	8–16	> 16
	Texture	L, SCL, SC, SiL, SiCL, CL, Si	SL, LS, LfS	LcS, fS, S, CS	Cm, SiCm
	Soil depth (cm)	> 75	75 − 50	50 − 20	< 20
	CaCO_3_%	< 12	12–20	20–25	> 25
	pH	5.8–7.6	7.6-8.0	8.0-8.2	> 8.2
	EC_e_ (dS/m)	< 1.5	1.5–2.5	2.5–3.5	> 3.5
Maize	Slope %	< 4	4–8	8–16	> 16
	Texture	C, SiC, SiCL, Si, SiL, CL, SC, L, SCL	SL, LS, LfS	fS, LcS, cS	Cm, SiCm
	Soil depth (cm)	> 75	75 − 50	50 − 20	< 20
	CaCO_3_%	0–15	15–25	25–35	> 35
	pH	6.5-8	8-8.2	8.2–8.5	> 8.5
	EC_e_ (dS/m)	< 4	4–6	6–8	> 8
Soybean	Slope %	< 4	4–8	8–16	> 16
	Texture	C < 60s, SiC, Co, SiCL, Si, SiL, CL, SC, C < 60v, C > 60s, L, SCL, C < 60v	C > 60v SL, LfS, LS	S, fS, LcS, cS	Cm, SiCm,
	Soil depth (cm)	> 75	75 − 50	50 − 20	< 20
	CaCO_3_%	< 15	15–20	20–25	> 25
	pH	6.5–7.5	7.5-8.0	8.0-8.5	> 8.5
	EC_e_ (dS/m)	< 6	6–8	8–10	> 10
Sugar beet	Slope %	< 4	4–8	8–16	> 16
	Texture	C < 60s, SiC, Co, SiCL, Si, SiL, SC, C < 60v, C > 60s, L, SCL,	C > 60v, SL, LfS, LS	S, fS, LcS	Cm, SiCm, cS
	Soil depth (cm)	> 50	50 − 20	20 − 10	< 10
	CaCO_3_%	< 30	30–45	45–75	> 75
	pH	6.5–8.2	8.2–8.3	8.3–8.5	> 8.5
	EC_e_ (dS/m)	< 8	8–12	12–16	> 16
Wheat	Slope %	< 4	4–8	8–16	> 16
	Texture	C < 60s, SiC, Co, Si, SiL, CL, C < 60v, SC, C > 60s, L,	C > 60v, SCL	SL, LfS, LcS, fS, cS	Cm, SiCm
	Soil depth (cm)	> 50	50 − 20	20 − 10	< 10
	CaCO_3_%	< 30	30–40	40–60	> 60
	pH	7.0-8.2	8.2–8.3	8.3–8.5	> 8.5
	EC_e_ (dS/m)	< 3	3–5	5–6	> 6

#### Analytical hierarchy process (AHP)

AHP is a systematic, multi-functional, and uncomplicated approach. It can manage a set of independent subjective and objective criteria under different decision-making circumstances. It enables the development of a hierarchy to meet the requirements of farmers or decision-makers^32,46^. Based on Saaty’s nine-point ratio scale, the AHP approach precisely measures the importance weight for each criterion for suitable land for major crop cultivation (Table 3)^47^. The sum of the importance weights of each hierarchy group is equal to 1. A scale of 1 indicates equal importance, while a score of 9 indicates that one factor is more important than the other. The reciprocals of 1 to 9 (1/1 and 1/9) demonstrate that one is less significant than the other^17,21,48^. The weights of each criterion in the pairwise comparison matrices were obtained using the Microsoft Excel Sheets software according to the previous literatures.

**Table 3:** Saaty scale for pairwise comparison between the criteria in the AHP.

AHP Scale of Importance for comparison pair	Numeric rating	Reciprocal
Extremely importance	9	1/9
Very strong to extremely	8	1/8
Very strong importance	7	1/7
Strongly to very strong	6	1/6
Strong importance	5	1/5
Moderately to strong	4	1/4
Moderate importance	3	1/3
Equally to moderately	2	1/2
Equal importance	1	1

Although literature’s’ statements are highly valued, there may be inconsistencies due to their preferences. As a result, Eq. 1 should be used to determine the Consistency Ratio (CR) to confirm the validity of the weights identified. CR is the ratio between the Consistency Index (CI) and the Random index (RI).

1 $${\text{CR}}\,=\,{\text{CI}}/{\text{RI}}$$

RI has been calculated for different n-values (number of criteria in the matrix) (Table 4). CI is the consistency index and can be defined by Eq. 2. Moreover, CI functions as a measure of logical inconsistency against literatures standards through peer-to-peer comparisons.

2 $${\text{CI }}={\text{ }}(lmax - n)/{\text{ }}\left( {n - \,{\text{1}}} \right)$$

Where, *λmax* is the maximum eigenvalue, and *n* is the total number of criteria in the comparison matrix.

The closer the *λmax* to the number (*n*) of the binary comparison matrix, the more consistent the results will be^31^. Moreover, CR should be ≤ 0.1 (less than 10% inconsistency) to be acceptable. Otherwise, the matrix should be changed^25^.

**Table 4:** Random inconsistency indices (RI).

*n*	1	2	3	4	5	6	7	8	9	10
RI	0	0	0.52	0.89	1.11	**1.25**	1.35	1.40	1.45	1.49

#### Weighted overlay of thematic maps

A weighted overlay analysis using ArcGIS Pro 3.0 was carried out with standardized raster-based maps and weighted importance for each criterion. Finally, each raster-based layer was integrated using Eq. 3^30^ to generate final output land suitability maps for major crop cultivation. The output raster-based layer was divided into four classes of equal interval according to the land suitability classification^4^, generated a land suitability map for major crop cultivation using ArcGIS Pro 3.0, and computed the class-wise areas.

3 $$\:\text{L}\text{S}\text{A}\:=\sum\nolimits_{i=1}^{n}Wi\:Si$$

Where, LSA is the final land suitability assessment, including S1, S2, S3, and N. $\:Wi$ is the weighted importance for the criterion *i*. $\:Si$ is the score of the criterion *i*. *n* is the total number of criteria for the assessment of land suitability.

## Results

###  Descriptive statistics for soil properties

**Fig. 4:**
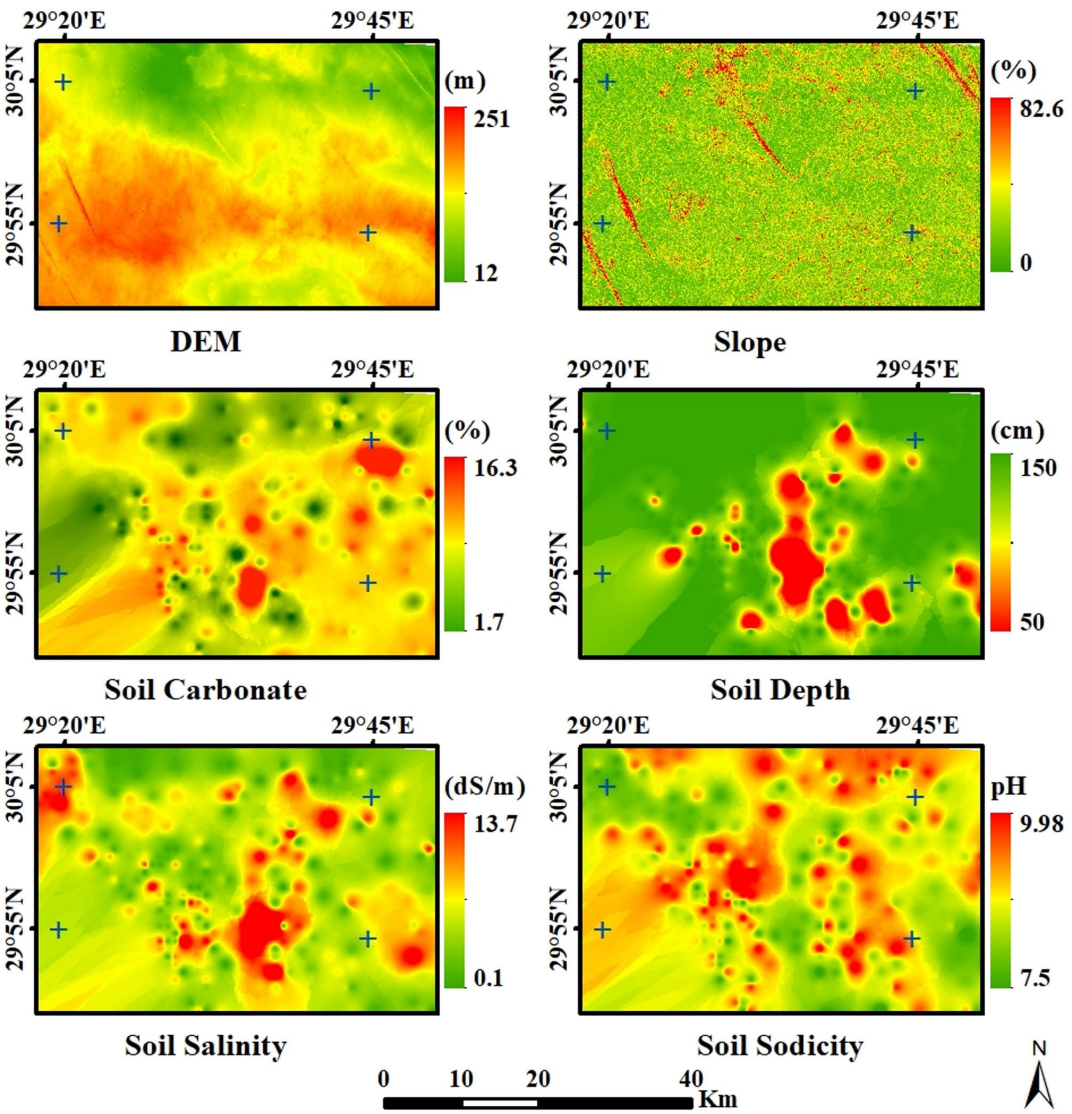
Soil properties of the study area.

With the IDW interpolation method, the results of the physical and chemical soil properties were shown on maps (Fig. 4), and the descriptive statistics analysis was shown in Table 5. The results indicated that the soil texture of the entire soil sample was mostly sand. The soil depth ranged between 50 and 150 cm, with an average depth of 142.65 cm. The majority of the area has deep soil that is suitable for various types of crops. pH values ranged between 7.5 and 9.9, with an average value of 8.7. Soil salinity varied from non-saline to saline, and EC values ranged between 0.1 and 13.76 dS/m with an average value of 1.8 dS/m. Soil calcium carbonate content ranged between 1.7% and 16.3%, with an average of 5.78%. The result obtained from the slope of the study area illustrated that 38.3% of the area was flat to almost flat (< 2%), 58.5% of the area was undulating (2–8%), 2.6% of the area was undulating to rolling (8–15%), 0.6% of the area was rolling to hilly (15–30%), and 0.1% of the area was greater than 30%. This indicated that about 96.8% of the study area was found to be very suitable for crop cultivation in terms of work efficiency and erosion control. In addition, the results indicated high variability for soil depth, pH, EC, and soil calcium carbonate content. Furthermore, a slight positive skewness (a normal distribution) was observed for pH, a high negative skewness for soil depth (skewed to deep soil depth values), and a high positive skewness for soil calcium carbonate content and EC (skewed to low values).

**Table 5:** Summary statistics of soil properties of the study area.

Statistics	Soil properties				
	Soil depth	pH	EC	CaCO_3_	Soil texture
Count	234	234	234	234	234
Min	50	7.49	0.1	1.69	-
Max	150	9.99	13.75	16.32	-
Mean	142.65	8.70	1.80	5.78	-
Stdev	20.06	0.69	1.74	2.10	-
CV ^1^	14.1	7.9	97.0	36.4	-
Skewness ^2^	-2.81	0.41	2.37	1.65	-
Kurtosis	6.98	-1.10	10.40	6.43	-

^1^ Coefficient of variation: <15 = low variation, 15–35 = moderate variation, > 35 = high variation.

^2^ Skewness: < |± 0.5| = normal distribution (symmetrical), ± 0.5–1.0 = moderately (positively or negatively) skewed of character, and > |± 1| highly (positively or negatively) skewed of character^21^.

###  Land suitability assessment results

The integration of AHP and GIS produced an extensive and sequential land suitability assessment for the production of barley, beans, maize, soybean, sugar beet, and wheat in the study area. The land suitability characteristics differed among crops depending on individual parameters which defined their evaluation.

#### Barley

The results indicated that slope was the controlling factor with a weighting of 39%. Very good places (S1) accounted for 28.4% of the study area with gentle slopes (< 8%), sandy soil, low salinity (EC < 12 dS/m), and soil depth > 50 cm. Moderately suitable (S2) and marginally suitable (S3) places accounted for 71% and 0.6%, respectively, following an increasing slope and salinity and decreasing soil depth.

#### Beans

Salinity (EC) was the governing parameter with a weighting of 41%. Very suitable areas (S1) were extremely minimal, only 0.2%, and included very low salinity areas (EC < 1.5 dS/m) and deep soil (> 75 cm). Moderately suitable (S2) areas were prevalent, covering 96.6% of the land, while marginally suitable (S3) areas were 3.2% owing to high salinity and shallow soil.

#### Maize

Slope was most significant, with a 35% weight. Most of the study area (77.2%) fell under highly suitable (S1) areas with gentle slopes (< 4%) and favorable soil characteristics. Moderately suitable (S2) areas were 22.82%, with steeper slopes and slightly higher salinity, and 0.01% fell under marginally suitable (S3) conditions.

#### Soybean

The evaluation found salinity (EC) was the most significant parameter, weighing at 41%. Very favorable sites (S1) were 2.2%, characterized by low salinity (EC < 6 dS/m) and deep soils (> 75 cm). Moderately favorable (S2) sites represented 97.8%, while marginally favorable (S3) sites were negligible (0.03%).

#### Sugar beet

Soil texture was the most important factor in sugar beet suitability with a weight of 36%. The entire research area was classified as moderately suitable (S2) on the basis of sandy soils, shallow depth (20–50 cm), and high salinity (EC 8–12 dS/m).

#### Wheat

Like barley, slope had the most significant effect on wheat suitability, at 39%. Extremely suitable places (S1) were extremely rare (0.04%), and moderately suitable (S2) places were predominant (92.1%). Marginally suitable (S3) places accounted for 7.9%, which was limited by steeper slopes, higher salinity, and shallow depth.

In summary, the AHP-GIS approach facilitated complete and precise assessment of land suitability for every crop, pointing out particular factors that had considerable influence on their cultivation capacity. The findings are beneficial to regional planning and long-run agriculture development in the target region.

Based on the results presented in Table 6, it was observed that slope emerged as the main factor influencing barley and wheat cultivation, exhibiting the largest specific weight (39%). Subsequently, texture, EC, pH, CaCO_3_, and depth were identified as significant factors, with specific weights of 25, 17, 10, 6, and 3, respectively. The EC factor emerged as the primary factor influencing bean and soybean cultivation, exhibiting the largest specific weight of 41%. Subsequently, pH, CaCO_3_, texture, depth, and slope were assigned specific weights of 24%, 17%, 9%, 5%, and 4%, respectively. The primary factor influencing maize cultivation was slope, followed by EC, pH, CaCO_3_, texture, and depth, with their respective weights of 35%, 28%, 16%, 10%, 6%, and 5%. The soil texture emerged as the primary factor for sugar beet cultivation, with depth, slope, EC, pH, and CaCO_3_ following suit. These factors were assigned precise weights of 25%, 19%, 11%, 6%, and 3% accordingly.

**Table 6:** Pairwise comparison matrix, importance weights, and consistency ratio of criteria in AHP for selected crops.

Barley	Pairwise comparison matrix						Weight	Rank	*n*	λ_max	Ci	RCI	CR
	Slope	Texture	EC	pH	CaCO_3_	Depth							
Slope	1	3	3	5	5	7	39	1	6	6.50	0.10	1.24	0.08
Texture	0.3	1	3	3	5	7	25	2					
EC	0.3	0.3	1	3	5	5	17	3					
pH	0.2	0.3	0.3	1	3	5	10	4					
CaCO_3_	0.2	0.2	0.2	0.3	1	3	6	5					
Depth	0.1	0.1	0.2	0.2	0.3	1	3	6					
Beans	EC	pH	CaCO_3_	Texture	Depth	Slope							
EC	1	3	3	5	7	7	41	1	6	6.45	0.09	1.24	0.07
pH	0.3	1	3	3	5	5	24	2					
CaCO_3_	0.3	0.3	1	3	5	5	17	3					
Texture	0.2	0.3	0.3	1	3	3	9	4					
Depth	0.1	0.2	0.2	0.3	1	3	5	5					
Slope	0.1	0.2	0.2	0.3	0.3	1	4	6					
Maize	Slope	EC	pH	CaCO_3_	Texture	Depth							
Slope	1	2	3	4	5	5	35	1	6	6.39	0.08	1.24	0.06
EC	0.5	1	3	3	5	7	28	2					
pH	0.3	0.3	1	3	3	5	16	3					
CaCO_3_	0.3	0.3	0.3	1	3	3	10	4					
Texture	0.2	0.2	0.3	0.3	1	3	6	5					
Depth	0.2	0.1	0.2	0.3	0.3	1	5	6					
Soybean	EC	pH	CaCO_3_	Texture	Depth	Slope							
EC	1	3	3	5	7	7	41	1	6	6.45	0.090	1.24	0.07
pH	0.3	1	3	3	5	5	24	2					
CaCO_3_	0.3	0.3	1	3	5	5	17	3					
Texture	0.2	0.3	0.3	1	3	3	9	4					
Depth	0.1	0.2	0.2	0.3	1	3	5	5					
Slope	0.1	0.2	0.2	0.3	0.3	1	4	6					
Sugar beet	Texture	Depth	Slope	EC	pH	CaCO_3_							
Texture	1	3	3	3	5	7	36	1	6	6.49	0.098	1.24	0.08
Depth	0.3	1	3	3	5	7	25	2					
Slope	0.3	0.3	1	3	5	7	19	3					
EC	0.3	0.3	0.3	1	3	5	11	4					
pH	0.2	0.2	0.2	0.3	1	3	6	5					
CaCO_3_	0.1	0.1	0.1	0.2	0.3	1	3	6					
Wheat	Slope	Texture	EC	pH	CaCO_3_	Depth							
Slope	1	3	3	5	5	7	39	1	6	6.5	0.10	1.24	0.08
Texture	0.3	1	3	3	5	7	25	2					
EC	0.3	0.3	1	3	5	5	17	3					
pH	0.2	0.3	0.3	1	3	5	10	4					
CaCO_3_	0.2	0.2	0.2	0.3	1	3	6	5					
Depth	0.1	0.1	0.2	0.2	0.3	1	3	6					

**Fig. 5:**
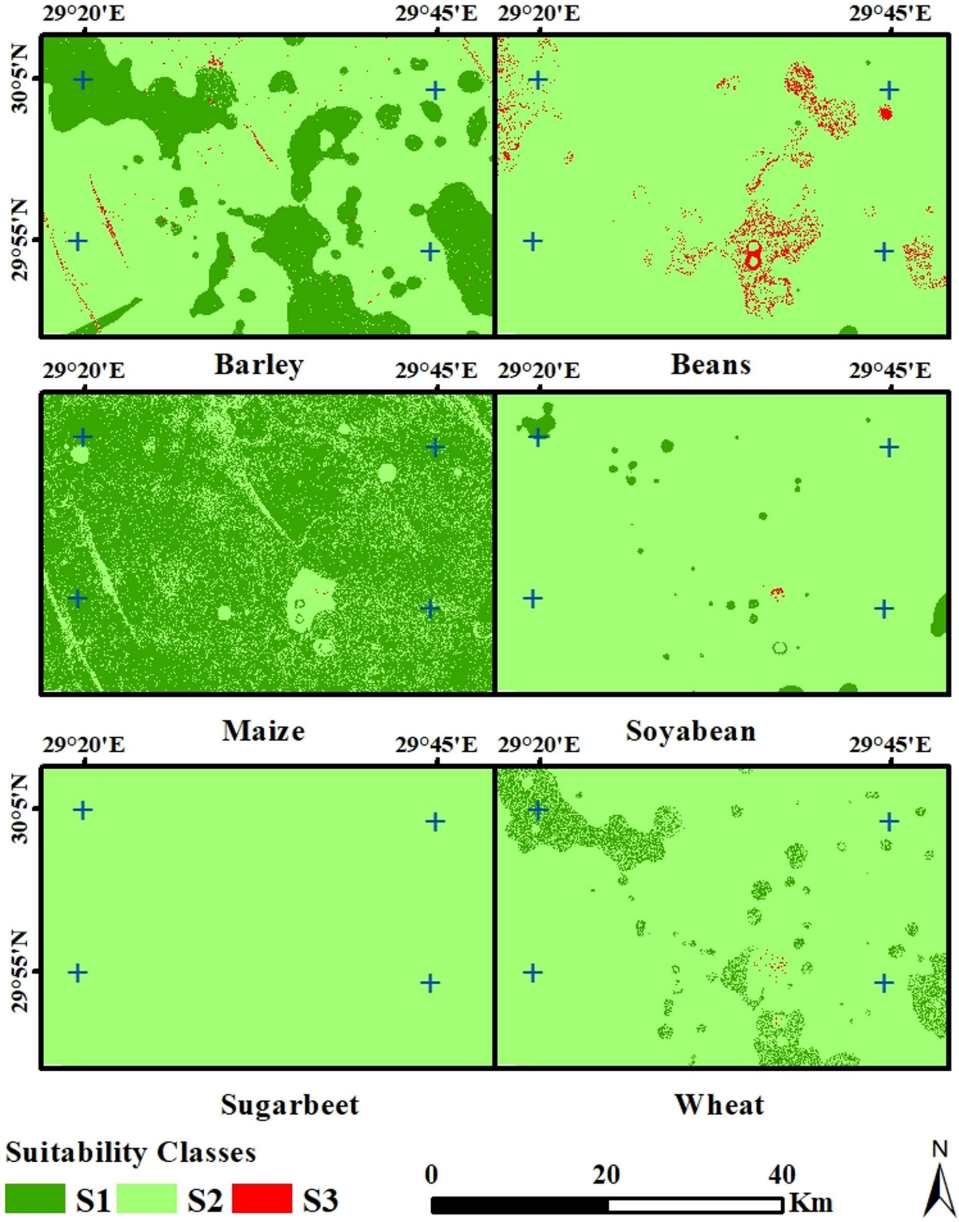
Land suitability classes for the tested crops in the study area.

The LSA maps of the selected crops revealed that land unit S1 for the barley map (Fig. 5) covers an area of 61650.97 hectares (28.4% of the total land area) (Table 7). This land unit is defined by a slope of less than 8%, a sandy soil texture, an EC of less than 12 dS/m, a pH range of 7.2 to 8.0, a CaCO_3_ content of less than 30%, and a soil depth greater than 50 cm (Table 2). The land unit S2 covers 154078.67 hectares (71% of the total area). It is characterized by slopes ranging from 8 to 12%, sandy soil texture, an EC of 12 to 16 dS/m, a pH of 8.0 at 8.2, a CaCO_3_ content of 30 to 40%, and soil depths ranging from 20 to 50 cm. Land unit S3 encompasses an area of 1272.20 hectares, accounting for 0.6% of the total land area. This land unit exhibits several characteristics, including slopes ranging from 12 to 16%, sandy soil texture, an EC ranging from 16 to 20 dS/m, a pH level of 8.2 at 8.5, a CaCO_3_ content ranging from 40 to 60%, and soil depths ranging from 10 to 20 cm.

**Table 7:** Land suitability classes areas (in hectares).

Crops	Area	Suitability classes			
		Highly suitable (S1)	Moderately suitable (S2)	Marginally suitable (S3)	Not suitable (N)
Barley	(ha)	61650.97	154078.67	1272.20	**-**
	%	28.4	71.0	0.6	**-**
Beans	(ha)	449.3	209675.6	6876.9	**-**
	%	0.2	96.6	3.2	**-**
Maize	(ha)	167464.3	49516.8	20.8	**-**
	%	77.17	22.82	0.01	**-**
Soybean	(ha)	4706.4	212224.6	70.9	**-**
	%	2.17	97.80	0.03	**-**
Sugar beet	(ha)	**-**	217001.8	**-**	**-**
	%	**-**	100	**-**	**-**
Wheat	(ha)	80.5	199767.5	17153.8	**-**
	%	0.04	92.06	7.90	**-**

According to the Beans map’s final LSA, land unit S1 has an area of 449.3 hectares (0.2%). This land unit is characterized by slopes of less than 4%, a sandy soil texture, an EC of less than 1.5 dS/m, a pH ranging from 5.8 to 7.6, CaCO_3_ levels below 12%, and a soil depth greater than 75 cm. The land unit S2 covers 209675.6 hectares, or 96.6% of the total land area. It is characterized by slopes ranging from 4 to 8%, sandy soil texture, EC from 1.5 to 2.5 dS/m, pH between 7.6 and 8.0, a CaCO_3_ concentration ranging from 12 to 20%, and soil depths ranging from 50 to 75 cm. Land unit S3 covers 6876.9 hectares, or 3.2% of the total land area. It is distinguished by slopes ranging from 8 to 16%, sandy soil texture, an EC of 2.5 to 3.5 dS/m, a pH of 8.0 at 8.2, a CaCO_3_ concentration ranging from 20 to 25%, and soil depths ranging from 20 to 50 cm.

The maize map’s final LSA revealed that land unit S1 encompasses an area of 167464.3 hectares, accounting for 77.17% of the total. This land unit is distinguished by slopes below 4%, a sandy soil texture, an EC below 4 dS/m, a pH ranging from 6.5 to 8.0, CaCO_3_ below 15%, and a soil depth beyond 75 cm. The land unit S2 covers 49516.8 hectares, or 22.82% of the total land area. It is characterized by slopes ranging from 4 to 8%, sandy soil texture, EC values ranging from 4 to 6 dS/m, pH levels ranging from 8.0 to 8.2, a CaCO_3_ concentration ranging from 15 to 25%, and soil depths ranging from 50 to 75 cm. The land unit S3 covers an area of 20.8 hectares (0.01%) and stands out due to its slopes ranging from 8 to 16%, sandy soil texture, an EC of 6 to 8 dS/m, a pH of 8.2 at 8.5, a CaCO_3_ content of 25 to 35%, and soil depths ranging from 20 to 50 cm.

The final LSA for the Soybean map revealed that land unit S1 covers an area of 4706.4 hectares (2.17%). This land unit is distinguished by slopes below 4%, a sandy soil texture, an EC below 6 dS/m, a pH ranging from 6.5 to 7.5, CaCO_3_ below 15%, and a soil depth greater than 75 cm. The land unit S2 spans an area of 212224.6 hectares (97.80%). It is characterized by slopes ranging from 4 to 8%, sandy soil texture, EC values ranging from 6 to 8.0 dS/m, pH levels ranging from 7.5 to 8.0, a CaCO_3_ concentration ranging from 15 to 20%, and soil depths ranging from 50 to 75 cm. The land unit S3 covers an area of 70.9 hectares (0.03%) and stands out due to its slopes ranging from 8 to 16%, sandy soil texture, an EC of 8 to 10 dS/m, a pH of 8.0 between 8.5 and 8.0, a CaCO_3_ content of 20 to 25%, and soil depths ranging from 20 to 50 cm.

In the sugar beet map’s final LSA, land unit S1 stands out as the sole LSA unit with an area of 217001.8 hectares (100%). This land unit exhibits specific characteristics, including slopes below 4%, a sandy soil texture, an EC value below 3 dS/m, a pH range of 7 to 8.2, CaCO_3_ levels below 30%, and a soil depth exceeding 50 cm.

The wheat map’s final LSA revealed that land units S1, covering 80.5 hectares (0.04%), exhibited slopes below 4%, a sandy soil texture, an EC below 3 dS/m, a pH ranging from 7 to 8.2, CaCO_3_ below 30%, and a soil depth beyond 50 cm. Land units S2, spanning an area of 199767.5 hectares (92.1%), exhibited slopes ranging from 4 to 8%, sandy soil texture, EC values ranging from 3 to 5 dS/m, pH levels ranging from 8.2 to 8.3, a CaCO_3_ content ranging from 30 to 40%, and soil depths ranging from 20 to 50 cm. The land unit S3 covers an area of 17153.8 hectares (7.90%), characterized by slopes ranging from 8 to 16%, sandy soil texture, an EC of 5 to 6 dS/m, a pH of 8.3 at 8.5, a CaCO_3_ content ranging from 40 to 60%, and soil depths ranging from 10 to 20 cm.

Rotating different crops according to the user’s choice is one of the important elements in increasing production and improving soil fertility by planting the land with more than one crop in the same year, so crop rotation was proposed as shown in Table 8. The crops were selected based on the requirements of the relevant factors and the division of the crops in the pattern for winter and summer.

**Table 8:** Distribution for winter and summer season crops for crop rotation based on LSA.

		Winter								Summer			
Description	Class	Barley		Beans		Wheat		Sugar beet		Maize		Soybean	
		(ha)	%	(ha)	%	(ha)	%	(ha)	%	(ha)	%	(ha)	%
Highly suitable	S1	61650.97	28.4	449.3	0.2	80.5	0.04	-	-	167464.3	77.17	4706.4	2.17
Moderately suitable	S2	154078.67	71	209675.6	96.6	199767.5	92.06	217001.8	100	49516.8	22.82	212224.6	97.8
Marginally suitable	S3	1272.2	0.6	6876.9	3.2	17153.8	7.9	-	-	20.8	0.01	70.9	0.03

## Discussion

### Importance of land suitability assessment

Land suitability assessment is critical for developing countries that wish to attain the maximum sustainable agricultural output. The identification and correction of limiting factors such as salinity, alkalinity, and land slope are a critical element in improving the efficiency of agriculture and productivity.

### Advantages of the AHP-GIS approach

Integration of AHP-GIS was discovered to be effective in the evaluation of criteria weights and assessment of land suitability for the intended crops. With six primary criteria to evaluate, the method enables effective land use analysis as well as selection of optimal crop rotation strategies. AHP-GIS outperforms traditional methods in offering more realistic and rational assessments, particularly with data heterogeneity^24,31,49–53^.

### Restricting key factors and management

Major limitations identified in the study are salinity, alkalinity, soil texture, and slope. Salinity and alkalinity can be countered by irrigation and gypsum application^58,59^. In the same way, the effect of calcium carbonate on soil pH and nutrient uptake can be countered by sulfur amendments^60–63^. Sandy soils, though less ideal because of water and nutrient loss, can be remedied with fertilizers and organic matter^55,56,64–67^. Slope constraints are best managed by high-level irrigation techniques, such as sprinkler or drip irrigation^54^.

### Climatic and environmental challenges

Climate aridity, wind erosion, and sand dune encroachment are factors that cause agricultural constraints. Soil conservation and sustainable land management are essential in the management of land degradation and the preservation of ecosystem services.

### Land use diversification and planning implications

Different land units possess different levels of suitability for given crops. Management practices and conservation methods can be implemented on a customized basis to enhance land use. AHP-GIS-based MCDA tool was found to be essential in the production of credible LSA maps, identifying the contribution of soil properties in sustainable growth^68^. The maps are capable of providing significant information to land use planners to prevent degradation problems and maximize agricultural yield^68–72^.

### Promotion of crop rotation and sustainability

Cultivation of fibrous and root crops through crop rotation is beneficial in increasing the quality of the soil, efficiency of application of water to irrigate crops, and chemical and physical aspects of the soil. Spread of weeds, insects, and plant pathogens is also reduced by adopting this practice and hence the long-term sustainability^70–72^.

### Implications for agricultural development

ArcGIS spatial modeling greatly helped in preparing detailed soil and land suitability maps. With integration of new agro practices into advanced suitability models, the study showed excellent prospects for enhancing the land use of newly developed places^71–73^. In the future, more research is needed to specifically address environmental issues to render the land use sustainable and climate resilient.

## Conclusions

It has been established that the application of AHP-GIS techniques has considerably enhanced land suitability evaluation, offering an integrated and dynamic approach towards sustainable agriculture development in arid and semi-arid areas. The study confirmed that land suitability mapping was a highly significant management tool to ensure planned and sustainable exploitation of agricultural land according to their potential. The model developed in this paper accounts for crop rotation variation through spatial and temporal productivity and offers automatically transferable results for analogous climatic and environmental areas.

The findings indicate potentiality of the study area to be partially to moderately able to sustain agricultural activities because it necessitates direct intervention. Major climatic factors like sunshine, relative humidity, and temperature possess moderate limiting effects, whereas soil factors like salinity, alkalinity, and calcium carbonate content are major sources of constraint towards crop production. Targeted management practices such as the use of gypsum and sulfur, organic amendments, and precision irrigation practices have improved the utilization of previously marginal land. These practices are aimed at the potential of transforming non-agricultural lands into productive agricultural plains.

AHP-GIS method, where FAO, Sys et al., and AHP models are integrated with ArcGIS application for spatial analysis, enables structured evaluation of quantitative soil and land attributes. The process is successful in aiding decision-making in thematic as well as suitability mapping to allow policymakers and planners to formulate quality decisions concerning land use. Additionally, the process is adaptive and can be applied to any area of geography that experiences common environmental challenges.

The research focuses on crop rotation activity in soil health, conservation of resources, and resistance to weed, pest, and disease. Through the incorporation of sustainability and conservation, the research offers a model that may be applied towards the enhancement of land productivity as well as arresting dry ecosystem challenges. The introduced model is an effective agricultural model for development, which is rational in the application of natural resources and enhancing environmental sustainability.

In conclusion, the work presents a new, flexible, and science-tested methodology for land suitability evaluation. The study emphasizes the application of several management methods to increase land and soil quality and guarantee long-term agricultural production and sustainability in areas with limited resources.

## Data Availability

Data Availability Statement: data will be made available upon reasonable request from the corresponding author: maekaoud@narss.sci.eg; maekaoud@gmail.com; maekaoud@yahoo.com; maekaoud@hotmail.com (M.A.E.A.); Tel.: +20-1004781114 (M.A.E.A).

## Abbreviations

AHP: Analytic hierarchy process
ALOS: Advanced land observing satellite
CI: Consistency index
CR: Consistency ratio
DEM: Digital elevation model
FAO: Food and Agriculture Organization of the United Nations
GIS: Geographic information system
LSA: Land suitability assessment
m a.s.l.: Unit for meter above sea level
MCDA: Multi-criteria decision analysis
RI: Random index
RS: Remote sensing
SAGA: System for Automated Geoscientific Analysis
UN: United Nations

## References

[1] Kennedy, C. M. et al. Optimizing land use decision-making to sustain Brazilian agricultural profits, biodiversity and ecosystem services. *Biol. Conserv.* **204**, 221–230 (2016).

[2] Pimentel, D. & Burgess, M. Soil erosion threatens food production. *Agriculture* **3** (3), 443–463 (2013).

[3] Mostafiz, R. B., Noguchi, R. & Ahamed, T. Agricultural land suitability assessment using satellite remote Sensing-Derived Soil-Vegetation indices. *Land* **10** (2), 1–26 (2021).

[4] FAO. *A Framework for Land Evaluation* (1976).

[5] Molla, A., Nigussie, D., Bishaw, Z., Mulugeta, W. & Biradar, C. Integrated Multi-criteria land suitability evaluation and mapping for scaling malt barley varieties in Rain-Fed production areas of Ethiopia. *J. Agric. Sci.* **12**(11), 123–140. 10.5539/jas.v12n11p123 (2020).

[6] Halder, J.C. Land suitability assessment for crop cultivation by using remote sensing and GIS. *J. Geogr. Geol.* **5** (3), 65 (2013).

[7] Sys, I., Van-Ranst, E. & Debveye, J. *Land Evaluation. Part 1: Principles in Land Evaluation.and Crop Production Calculations* (General Administration for Development Cooperation, 1991). (Agricultural Publications No. 7).

[8] Saleh, A. M., Belal, A. B. & Mohamed, E. S. Land resources assessment of El-Galaba basin, South Egypt for the potentiality of agriculture expansion using remote sensing and GIS techniques. *Egypt. J. Remote Sens. Space Sci.* **18** (1), S19–S30 (2015).

[9] Esmail, M., Masria, A. L. I. & Negm, A. Monitoring land use/land cover changes around Damietta promontory, egypt, using RS/GIS. *Procedia Eng.* **154**, 936–942 (2016).

[10] Alnaimy, M. A., Soliman, K. G. & El-Naka, E. A. Evaluation of land suitability for agricultural use in El-Nubaria region, Egypt. *Zagazig J. Agricultural Res.* **45** (6), 2031–2048 (2018).

[11] Allani, M. et al. A contribution to an advisory plan for integrated irrigation water management at Nebhana dam system: from research to operational support. *EPiC Ser. Eng.* **3**, 26–15 (2018).

[12] Mohamed, S. A. & El-Raey, M. E. Vulnerability assessment for flash floods using GIS Spatial modeling and remotely sensed data in El-Arish city, North sinai, Egypt. *Nat. Hazards*. **102** (2), 707–728 (2020).

[13] Mendas, A. & Delali, A. Integration of multicriteria decision analysis in GIS to develop land suitability for agriculture: application to durum wheat cultivation in the region of Mleta in Algeria. *Comput. Electron. Agric.* **83**, 117–126 (2012).

[14] Feizizadeh, B. & Blaschke, T. Land suitability analysis for Tabriz county, iran: a multi-criteria evaluation approach using GIS. *J. Environ. Planning Manage.* **56** (1), 1–23 (2013).

[15] Elsheikh, R. et al. Agriculture land suitability evaluator (ALSE): A decision and planning support tool for tropical and subtropical crops. *Comput. Electron. Agric.* **93**, 98–110 (2013).

[16] Purnamasari, R. A., Noguchi, R. & Ahamed, T. Land suitability assessment for cassava production in Indonesia using GIS, remote sensing, and multi-criteria analysis. In *Remote Sensing Application*. 99–132. (Springer, 2022).

[17] Zhang, J., Sun, H., Jiang, X. & He, J. Evaluation of development potential of cropland in Central Asia. *Ecolo. Indic.* **142**, 109250 (2022).

[18] Saaty, T. L. *The Analytic Hierarchy Process: Planning, Priority Settling. Resource Allocation* (McGraw-Hill International Book Company, 1980).

[19] Mishra, A. K., Deep, S. & Choudhary, A. Identification of suitable sites for organic farming using AHP & GIS. *Egypt. J. Remote Sens. Space Sci.* **18** (2), 181–193 (2015).

[20] Aldababseh, A., Temimi, M., Maghelal, P., Branch, O. & Wulfmeyer, V. Multi-criteria evaluation of irrigated agriculture suitability to achieve food security in an arid environment. *Sustainability*, **10**(3), 803 (2018).

[21] Iliquín Trigoso, D. et al. M.Á., Land suitability analysis for potato crop in the Jucusbamba and Tincas Microwatersheds (Amazonas, NW Peru): AHP and RS–GIS approach. *Agronomy* **10**(12), 1898 (2020).

[22] Daylam, F., Kazemi, H. & Kamkar, B. Modelling organic farming suitability by spatial indicators of GIS integrated MCDA in Golestan Province, Iran. In *NJAS: Impact in Agricultural and Life Sciences*. Vol. 95(1). 2191796 (2023).

[23] Akıncı, H., Özalp, A. Y. & Turgut, B. Agricultural land use suitability analysis using GIS and AHP technique. *Comput. Electron. Agric.* **97**, 71–82 (2013).

[24] Zhang, J., Su, Y., Wu, J. & Liang, H. *GIS Based Land Suitability Assessment for Tobacco Production Using AHP and Fuzzy Set in Shandong Province of China*. Vol. 114. 202–211 (Computers and Electronics in Agriculture, 2015).

[25] Ramamurthy, V., Reddy, G. O. & Kumar, N. Assessment of land suitability for maize (*Zea mays* L) in semi-arid ecosystem of southern India using integrated AHP and GIS approach. *Comput. Electron. Agric.* **179**, 105806 (2020).

[26] Soil Survey Staff. Kellogg soil survey laboratory methods manual. In *Soil Survey Investigations Report No. 42, Version 5.0*. (2014).

[27] AbdelRahman, M. A., Zakarya, Y. M., Metwaly, M. M. & Koubouris, G. Deciphering soil spatial variability through geostatistics and interpolation techniques. *Sustainability*, **13**(1), 194 (2020).

[28] Jenny, H. *Factors of Soil Formation: A System of Quantitative Pedology* (Courier Corporation, 1994).

[29] Zolekar, R. B. & Bhagat, V. S. Multi-criteria land suitability analysis for agriculture in hilly zone: remote sensing and GIS approach. *Comput. Electron. Agric.* **118**, 300–321 (2015).

[30] Ostovari, Y. et al. GIS and multi-criteria decision-making analysis assessment of land suitability for rapeseed farming in calcareous soils of semi-arid regions. *Ecol. Ind.* **103**, 479–487 (2019).

[31] Dedeoğlu, M. & Dengiz, O. Generating of land suitability index for wheat with hybrid system aproach using AHP and GIS. *Comput. Electron. Agric.* **167**, 105062 (2019).

[32] Tercan, E. & Dereli, M. A. Development of a land suitability model for citrus cultivation using GIS and multi-criteria assessment techniques in Antalya Province of Turkey. *Ecol. Indic.*, **117**, 106549 (2020).

[33] Tesfay, T., Biedemariam, M., Hagazi, M. & Gebretinsae, T. Land capability and suitability evaluation for rain-fed crops in semi-arid lowland area of North Ethiopia. *Vegetos—An Int. J. Plant. Res.* **30** (3), 18–22 (2017).

[34] Griffel, L. M. et al. A multi-criteria land suitability assessment of field allocation decisions for switchgrass. *Ecol. Indic.* **136**, 108617 (2022).

[35] Klingebiel, A. A. & Montgomery, P. H. Land capability classification, agricultural handbook. (Soil Conservation Service, United States Department of Agriculture, 1961).

[36] FAO. A framework for land evaluation. *Int. Inst. Land. Reclam. Improv.* **22**, 87 (1977).

[37] De la Rosa, D., Cardona, F. & Almorza, J. Crop yield predictions based on properties of soils in sevilla, Spain. *Geoderma* **25** (3–4), 267–274 (1981).

[38] Mueller, L. et al. Assessing the productivity function of soils. A review. *Agron. Sustain. Dev.* **30** (3), 601–614 (2010).

[39] Richards, L. A. Diagnosis and improvement of saline and alkali soils. Vol. 78(2). 154. LWW. (United States Department of Agriculture, 1954).

[40] Salas López, R. et al. Land suitability for coffee (Coffea arabica) growing in Amazonas, Peru: Integrated use of AHP, GIS and RS. *ISPRS Int. J. Geo-Inf.* **9**(11), 673 (2020).

[41] Rhoades, J. D., Kandiah, A. & Mashali, A. M. *The Use of Saline Waters for Crop Production. FAO Irrigation and Drainage Paper 48* (Food and Agriculture Organization of the United Nations, 1992).

[42] Sys, I. C., van Ranst, E. & Debaveye, I. J. *Land Evaluation. Part III: Crop Requirements. International Training Center (ITC) for Post-Graduate Soil Scientists University Gent, Agricultural Publications No. 7* (General Administration for Development Cooperation, 1993).

[43] Fipps, G. *Irrigation Water Quality Standards and Salinity Management. Fact Sheet B-1667. Texas Cooperative Extension* (The Texas A&M University System, 2003).

[44] Zakarya, Y. M. Thesis Land Resources Assessment Using GIS, Expert Knowledge and Remote Sensing in the Desert Environment. (Ph.D. Department of Soil Science, Faculty of Agriculture, Ain Shams University, 2009).

[45] Metwaly, M. M. & Thesis, P. D. Sustainable Land Use Planning of El-Qaa Plain, South Sinai, Egypt. (Department of Soil Science, Faculty of Agriculture, Ain Shams University, 2013).

[46] Veisi, H., Liaghati, H. & Alipour, A. Developing an ethics-based approach to indicators of sustainable agriculture using analytic hierarchy process (AHP). *Ecol. Ind.* **60**, 644–654 (2016).

[47] Saaty, T. L. & Vargas, L. G. How to make a decision. In: Models, Methods, Concepts & Applications of the Analytic Hierarchy Process. International Series in Operations Research & Management Science. Vol 175. 10.1007/978-1-4614-3597-6_1 (Springer, 2012).

[48] Pilevar, A. R., Matinfar, H. R., Sohrabi, A. & Sarmadian, F. Integrated fuzzy, AHP and GIS techniques for land suitability assessment in semi-arid regions for wheat and maize farming. *Ecol. Indic.*, **110**, 105887. (2020).

[49] Kılıc, O. M., Ersayın, K., Gunal, H., Khalofah, A. & Alsubeie, M. S. Combination of fuzzy-AHP and GIS techniques in land suitability assessment for wheat (Triticum aestivum) cultivation. *Saudi J. Biol. Sci.* **29** (4), 2634–2644 (2022). 35531232 10.1016/j.sjbs.2021.12.050 PMC9073045

[50] Zhang, S., Liu, X., Wang, X., Gao, Y. & Yang, Q. Evaluation of coffee ecological adaptability using fuzzy, AHP, and GIS in Yunnan province, China. *Arab. J. Geosci.* **14** (14), 1–18 (2021).

[51] Bagheri, M. et al. A., Land-use suitability assessment using Delphi and analytical hierarchy process (D-AHP) hybrid model for coastal city management: Kuala Terengganu, Peninsular Malaysia. *ISPRS Int. J. Geo-Inf.* **10**(9), 621 (2021).

[52] Jaroenkietkajorn, U. & Gheewala, S. H. Land suitability assessment for oil palm plantations in Thailand. *Sustainable Prod. Consum.* **28**, 1104–1113 (2021).

[53] Özkan, B., Dengiz, O. & Turan, İ. D. Site suitability analysis for potential agricultural land with Spatial fuzzy multi-criteria decision analysis in regional scale under semi-arid terrestrial ecosystem. *Sci. Rep.* **10** (1), 1–18. 10.1038/s41598-020-79105-4 (2020). 33328573 10.1038/s41598-020-79105-4 PMC7744537

[54] Rossiter, D. G. A theoretical framework for land evaluation. *Geoderma* **72** (3–4), 165–190 (1996).

[55] Seyedmohammadi, J., Sarmadian, F., Jafarzadeh, A. A. & McDowell, R. W. Development of a model using matter element, AHP and GIS techniques to assess the suitability of land for agriculture. *Geoderma* **352**, 80–95 (2019).

[56] Seyedmohammadi, J., Sarmadian, F., Jafarzadeh, A. A. & McDowell, R. W. Integration of ANP and fuzzy set techniques for land suitability assessment based on remote sensing and GIS for irrigated maize cultivation. *Arch. Agron. Soil. Sci.* **65** (8), 1063–1079 (2019).

[57] Tashayo, B., Honarbakhsh, A., Azma, A. & Akbari, M. Combined fuzzy AHP–GIS for agricultural land suitability modeling for a watershed in Southern Iran. *Environ. Manage.* **66** (3), 364–376 (2020). 10.1007/s00267-020-01310-8 32533327

[58] Abdel-Fattah, M. K. Role of gypsum and compost in reclaiming saline-sodic soils. *J. Agric. Vet. Sci.* **1** (3), 30–38 (2012).

[59] Silveira, K. R. D., Ribeiro, M. R., Oliveira, L. B. D., Heck, R. J. & Silveira, R. R. D. Gypsum-saturated water to reclaim alluvial saline sodic and sodic soils. *Scientia Agrícola*. **65**, 69–76 (2008).

[60] Diacono, M. & Montemurro, F. Long-term effects of organic amendments on soil fertility. In Sustainable Agriculture. Vol. 2. 761–786. (Springer, 2011).

[61] Barka, H. A. F., Benzaghta, M. A. & Kasheem, A. M. Effect of different organic matters on chemical properties of calcareous soil. *Sirte Univ. Sci. J.* **8** (2), 101–110 (2018).

[62] Mustafa, A. A. et al. Land suitability analysis for different crops: a multi criteria decision making approach using remote sensing and GIS. *Researcher* **3** (12), 61–84 (2011).

[63] Taalab, A. S., Ageeb, G. W., Siam, H. S. & Mahmoud, S. A. Some characteristics of calcareous soils. A review AS Taalab1, GW Ageeb2, Hanan S. Siam1 and Safaa A. Mahmoud1. *Middle East. J.* **8** (1), 96–105 (2019). http://www.curresweb.com/mejar/mejar/2019/96-105.pdf

[64] Warrence, N. J., Bauder, J. W. & Pearson, K. E. Basics of salinity and sodicity effects on soil physical properties. *Department of Land Resources and Environmental Sciences, Montana State University-Bozeman, MT*, *129*, pp.1–29. (2002).

[65] Seyedmohammadi, J., Esmaeelnejad, L. & Ramezanpour, H. Land suitability assessment for optimum management of water consumption in precise agriculture. *Model. Earth Syst. Environ.* **2** (3), 1–11 (2016).

[66] Seyedmohammadi, J., Sarmadian, F., Jafarzadeh, A. A., Ghorbani, M. A. & Shahbazi, F. Application of SAW, TOPSIS and fuzzy TOPSIS models in cultivation priority planning for maize, rapeseed and soybean crops. *Geoderma* **310**, 178–190 (2018).

[67] Kaledhonkar, M. J., Meena, B. L. & Sharma, P. C. Reclamation and nutrient management for salt-affected soils. *Indian J. Fertilisers*. **15** (5), 566–575 (2019).

[68] D’Antonio, P. et al. Modeling climatic, terrain and soil factors using AHP in GIS for grapevines suitability assessment. *Sustain. Dev*. 1–22. 10.1002/sd.3136 (2024).

[69] Saleh, A. M., Elsharkawy, M. M., AbdelRahman, M. A. E. & Arafat, S. M. Evaluation of soil quality in arid western fringes of the Nile Delta for sustainable agriculture. *Appl. Environ. Soil Sci*. **1434692**, 17 (2021). 10.1155/2021/1434692

[70] AbdelRahman, M. A. E. et al. Mapping of soils and land-related environmental attributes in modern agriculture systems using geomatics. *Sustain. Water Resour. Manag*. **8**, 116. 10.1007/s40899-022-00704-2 (2022).

[71] AbdelRahman, M. A. E., Saleh, A. M. & Arafat, S. M. Assessment of land suitability using a soil-indicator-based approach in a geomatics environment. *Sci. Rep.* **12**, 18113. 10.1038/s41598-022-22727-7 (2022). 36302834 10.1038/s41598-022-22727-7 PMC9613761

[72] AbdelRahman, M. A. E. & Arafat, S. M. An approach of agricultural courses for soil conservation based on crop soil suitability using geomatics. *Earth Syst. Environ.* **4**, 273–285. 10.1007/s41748-020-00145-x (2020).

[73] AbdelRahman, M. A. E., Shalaby, A. & Mohamed, E. S. Comparison of two soil quality indices using two methods based on geographic information system. *Egypt. J. Remote Sens. Space Sci.* **22** (2), 127–136. 10.1016/j.ejrs.2018.03.001 (2019). 2018.

